# Children who develop type 1 diabetes early in life show low levels of carnitine and amino acids at birth: does this finding shed light on the etiopathogenesis of the disease?

**DOI:** 10.1038/nutd.2013.33

**Published:** 2013-10-28

**Authors:** G la Marca, S Malvagia, S Toni, B Piccini, V Di Ciommo, G F Bottazzo

**Affiliations:** 1 Newborn Screening, Biochemistry and Pharmacology Labs, Clinic of Pediatric Neurology, A Meyer Children's Hospital, Florence, Italy; 2 NeuroFarba Department, University of Florence, Florence, Italy; 3 Juvenile Diabetes Center, A Meyer Children's Hospital, Florence, Italy; 4 Unit of Epidemiology, Department of Medical Directorate, Bambino Gesù Children's Hospital, Rome, Italy; 5 The Bottazzo's House, San Marco 3315, Venice, Italy

**Keywords:** carnitines, amino acids, thymic central tolerance, type 1 diabetes

## Abstract

**Background:**

Children and adolescents with overt type 1 diabetes (T1D) have been found to show an altered carnitine profile. This pattern has not previously been analyzed in neonates before onset of the disease.

**Materials and methods:**

Fifty children who developed T1D during the first 6 years of life, born and living in the Tuscany and Umbria Regions of Italy, were identified and 200 controls were recruited into the study. All newborns were subjected to extended neonatal screening by mass spectrometry at 48–72 h of life. Four controls for each of the 50 index cases were taken randomly and blinded in the same analytical batch. The panel used for neonatal screening consists of 13 amino acids, free carnitine, 33 acyl-carnitines and 21 ratios. All Guthrie cards are analyzed within 2 days of collection.

**Results:**

Total and free carnitine were found to be significantly lower in neonates who later developed T1D compared with controls. Moreover, the concentrations of the acyl-carnitines – acetyl-L-carnitine (C2), proprionylcarnitine (C3), 3-hydroxyisovalerylcarnitine (C5OH), miristoylcarnitine (C4), palmitoylcarnitine (C16) and stearoylcarnitine (C18) – were also significantly low in the cases vs controls. Furthermore, total amino-acid concentrations, expressed as the algebraic sum of all amino acids tested, showed a trend toward lower levels in cases vs controls.

**Conclusions:**

We found that carnitine and amino-acid deficit may be evident before the clinical appearance of T1D, possibly from birth. The evaluation of these metabolites in the neonatal period of children human leukocyte antigen genetically at ‘risk' to develop T1D, could represent an additional tool for the prediction of T1D and could also offer the possibility to design new strategies for the primary prevention of the disease from birth.

## Introduction

The mechanisms involved in the triggering of autoimmune aggression against beta cells, which leads to clinically overt type 1 diabetes (T1D), are still poorly understood, but a complex interaction between genetic,^1^ immunologic,^2^ environmental^3^ and epigenetic^4^ factors has been repeatedly postulated. Several prospective studies have demonstrated that factors operating early in life, and even in utero, may have an important role in the etiopathogenesis of T1D and/or may explain why certain individuals have an increased risk of developing the disease. These factors include dietary elements, such as short breast feeding or early exposure to cow's milk^5^ as well as the early introduction of gluten into the diet,^6^ all of which possibly interfere with carnitine and amino-acid metabolism.

A reduced pool of free carnitine has been reported in children with overt T1D.^7, 8^ Based on these data, we performed a case-control study aimed at investigating the metabolic profiles of carnitines and amino acids in the first days of life and their possible relationship with the future development of T1D. We indeed found that 50 neonates, who progressed to develop T1D, had significantly low levels of total carnitine, free carnitine and acyl-carnitines, compared with levels detected in 200 controls. In addition, a trend of low levels of amino acids was detected in the same neonates. Information will also be provided on the 6-year follow-up of the index cases and of the controls subsequently enrolled in the study.

The data we have produced indicate that the carnitine and amino-acid deficit may impede the ‘negative selection' of potentially autoreactive T cells, a process which occurs in the first days of life in the medulla of the thymus and leads to the death of the T cells by apoptosis. As a result, the autoreactive T cells escape death by apoptosis, leave the thymus and become ‘drifting mines' in the peripheral lymphoid organs, ready, if activated, to attack and ultimately kill beta cells in the pancreatic islets. In view of our data, strategies could be designed for the primary prevention of T1D.

## Materials and methods

### Study population

We enrolled 50 children (26 female and 24 male subjects) with T1D diagnosed between November 2006 and May 2012 and/or followed at Meyer Children's Hospital during the same period. The children were born between January 2005 and April 2010 in the Tuscany or Umbria Regions of Italy where an expanded neonatal screening was performed on dried blood spots (DBS). We excluded children with transient or permanent neonatal diabetes and children with monogenic diabetes. In addition, we excluded low weight (<1800 g) pre-term newborns, newborns on parenteral nutrition and newborns receiving an exsanguino-transfusion before the newborn screening test. Controls consisted of 200 neonates, 4 for each of the 50 index cases taken randomly and blinded in the same analytic batch and born around the same day (+/− 1 day). All experiments were conducted in compliance with the Institutional Review Board guidelines of the Meyer Children's Hospital Ethic Committee which approved this study (protocol no. *234/2013*).

### Clinical assessment

T1D was diagnosed according to the American Diabetes Association (ADA) criteria:^9^ (1) glycated hemoglobin (HbA1c) ⩾6.5% (in accordance with the National Glycohemoglobin Standardization Program certified and standardized to the Diabetes Control and Complication Trial assay); (2) fasting plasma glucose ⩾126 mg dl^−1^ (7.0 mmol l^−1^). Fasting is defined as no caloric intake for at least 8 h; (3) 2-h plasma glucose ⩾200 mg dl^−1^ (11.1 mmol l^−1^) during an oral glucose tolerance test. The test should be performed as described by the World Health Organization, using a glucose load containing the equivalent of 75 g anhydrous glucose dissolved in water; (4) In patients with classic symptoms of hyperglycemia or hyperglycemic crisis, a random plasma glucose ⩾200 mg dl^−1^ (11.1 mmol l^−1^).

### Islet-related-antibodies (I-r-Abs) determination

At diagnosis, 44 diabetic children were tested for the presence of islet-related-antibodies (I-r-Abs), in particular, ICA (islet cell antibodies; ICA 5 or >JDFU detected by indirect immune-fluorescence), IAA (insulin autoantibodies; IAA >1.5, radioimmunoassay method), GADA (glutamic acid decarboxylase antibodies; GADA >0.75, radioimmunoassay method); insulinoma-associated antigen-2 antibodies (IA-2A >0.75 up to 31 December 2011; IA-2A >5 from 01 January 2012, radioimmunoassay method).

### HLA typing

At diagnosis, 29 diabetic children were human leukocyte antigen (HLA) typed using *PCR*-*SSP* (*polymerase chain reaction*-specific sequence primers; Kit-*BAG*, *Germany)*.^10^

### Metabolite profiling

The Expanded Newborn Screening Pilot Programme using liquid chromatography–tandem mass spectrometry (LC-MS/MS) has been operating in three provinces of Tuscany since January 2002. Officially mandated by legislative action, the programme has screened all babies born in Tuscany since November 2004 (∼35 000 per year) and all babies born in Umbria since 2010 (∼7500 per year) for selected acyl-carnitines and amino acids.^11, 12, 13^

*Materials*: Labeled internal standards of acyl-carnitines and amino acids were purchased from Cambridge Isotope Laboratories (Andover, MA, USA); a stock solution was made in methanol. The standard concentrations were in the range of 500–2500 μmol l^−1^ for amino acids, and in the range of 7.6–152 μmol l^−1^ for acyl-carnitines. Daily working solutions were made by dilution of the stock solution (1:200) using methanol–water 90:10 v/v. External standard blood spots for acyl-carnitines and amino acids from CDC (Atlanta, GA, USA) were run every day for each instrument. Routine MS/MS analysis is performed on two instruments (API 4000 AB SCIEX, Toronto, Canada) and one additional instrument (API 2000 AB SCIEX) for back up. The analyte concentrations are calculated automatically using the Chemoview software (API 4000 AB SCIEX). The analyte panel used for neonatal screening consists of 13 amino acids, free carnitine, 33 acyl-carnitines and 21 ratios. Guthrie card collection procedure DBS samples are routinely collected from all neonates born in the Tuscany and Umbria Regions (about 42 000 per year), as reported by la Marca and colleagues.^13^ Briefly blood collection (eight DBS on a Guthrie card) is recommended between 48 and 72 h after delivery. Blood samples are obtained by heel stick, spotted on filter paper (903, Whatman GmbH, Dasel, Germany), dried and sent daily by courier to the central newborn screening laboratory. An expanded newborn screening is routinely performed and includes hypothyroidism, cystic fibrosis, biotinidase deficiency and over 40 inborn errors of metabolism of amino acids, organic acids and fatty acids. A pilot project for ADA SCID started in 2011 for the entire neonatal population.^14^ All Guthrie cards are analyzed within 2 days of collection; after use, all cards are stored and, if not used, discarded after 10 years.

*Tandem mass spectrometry*. An AB SCIEX API 4000 bench-top Triple-Quad Mass Spectrometer equipped with the TurboIonSpray source was used for this study. The TurboIonSpray source operated under positive ion mode at a voltage of 5500 volts and with a ‘turbo' gas flow of 10 l min^−1^ of air heated to 450 °C (nominal heating-gun temperature). Mass calibration and resolution adjustments on the resolving quadrupoles were performed automatically using a PPG 2 × 10-6 mol l^−1^ solution introduced via the built-in Infusion Pump. The resolution was set on both resolving quadrupoles at 0.7 amu (measured at 1/2 height) for all MS and MS/MS experiments.

Collision-activated dissociation MS/MS was performed through the LINAC Q2 collision cell, operating with 10 mTor pressure of nitrogen as collision gas. Declustering potential, collision exit potential and collision energy were automatically optimized for both unlabeled amino acids and acyl-carnitines by the ‘quantitation optimization' option. The resulting declustering potential was +50 V, optimal collision energy and collision exit potential were seen at 26 V and 20 V, respectively.

### Statistical analysis

Data were preliminarily tested for normality with K–Smirnov test, and descriptive data were accordingly expressed as mean±s.d., or median with interquartile range. Comparisons between means were performed with Student's *t*-test and comparisons between medians with the Mann–Whitney test. SPSS software (13.0) was used and a *P*<0.05 considered statistically significant.

## Results

### Clinical characteristics of the children with T1D

The 50 children enrolled in the study showed classical signs and symptoms of T1D such as polyuria, polydipsia, polyphagia, enuresis, fatigue, weight loss and dehydration. At diagnosis of diabetes, the average age was 3 years and 1 month (range 8 months to 6 years). At T1D onset, the average blood glucose level was 574 mg dl^−1^ (31.8 mmol l^−1^; range 202–1243 mg dl^−1^), average HbA1c level was 10.9% (range 7.3–14%). Of the 50 diabetic children, 20% showed severe diabetic ketoacidosis, defined as blood pH lower than 7.1 and bicarbonate levels lower than 5 mmol l^−1^. Of the 50 index cases, 33 developed T1D between 0.8 and 3.8 years and 15 between 4.1 and 6.5 years. As for the 50 index cases, none of the 200 control neonates had T1D at the time of enrollment in the study and none developed T1D in the 6 years of follow-up.

### Associated diseases in the T1D children and their relatives

Celiac disease (CD) was diagnosed in 11 children after T1D onset, whereas in one child CD preceded T1D onset. None of the index cases had autoimmune thyroid or other autoimmune diseases. Six children had first-degree relatives with T1D: four fathers, one mother and one sister. In addition, one father had Graves' disease, one mother had CD and two children had second-degree relatives with T1D (two grandmothers) (Table 1).

### Islet-related-antibodies

ICA, IAA, GADA and IA-2A were detected in 44 of the T1D children (at least one I-r-Abs), 2 children had no detectable I-r-Abs and in 4 cases I-r-Abs were not tested (Table 1).

### HLA typing

We found HLA genetic susceptibility markers for T1D (DRB1*03/DRB1*04-DQB1*0302) in 27 of the 29 HLA-typed children (Table 1). Of the 50 children with T1D, 25 had both detectable I-r-Abs and HLA genetic susceptibility to T1D, 17 had detectable I-r-Abs, but were not typed for HLA, 2 had HLA genetic susceptibility to T1D, but did not have detectable I-r-Abs, 2 had detectable I-r-Abs, but did not have HLA genetic susceptibility to T1D and 4 were neither tested for I-r-Abs nor HLA typed (Table 1).

### Levels of carnitines and amino acids at birth in neonates at ‘risk' to develop T1D

**Figure 1:**
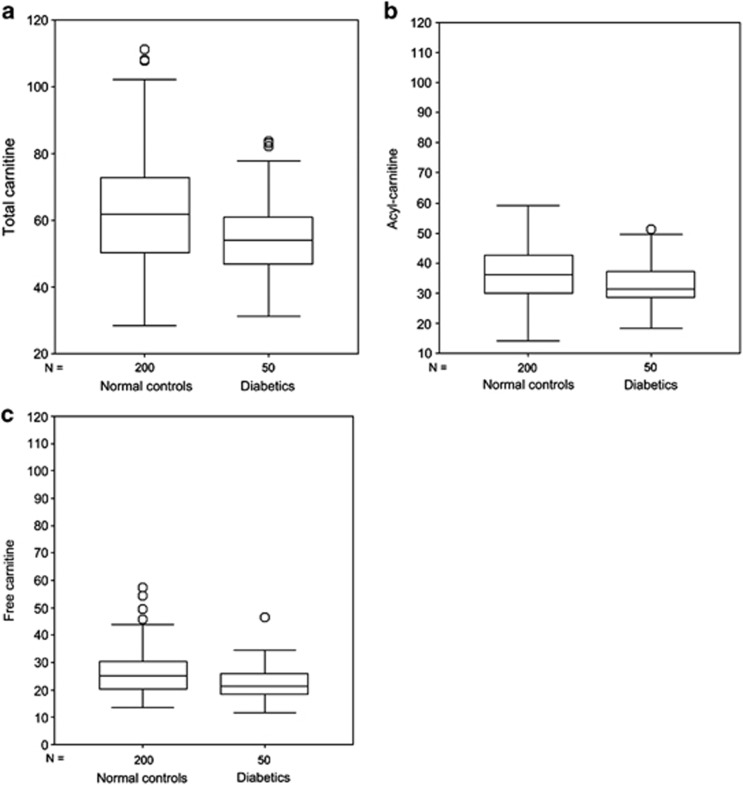
Box-plot of total carnitine (**a**), acyl-carnitine (**b**) and free carnitine (**c**) in newborns who developed T1D and in controls. The horizontal line across the box represents the median, and the box comprises 50% of the patients, an half lower and half higher than the median; vertical lines represent about the higher and the lower 25% of the patients (circles are the extreme values).

Compared with controls, significantly low levels of total carnitine, free carnitine and acyl-carnitines were found in neonates who would develop T1D later in their childhood. The differences in the levels of carnitine between cases and controls showed high statistical significance (Figures 1a–c), whereas the 21 acyl/free carnitine ratios were only marginally significant (*P*=0.07) (Table 2). In addition, levels of 28 acyl-carnitines were approximately the same in index cases vs controls (data not shown), while the acyl-carnitines – acetyl-L-carnitine (ALCAR) (C2), proprionylcarnitine (C3), hydroxyisovalerylcarnitine (C5OH), miristoylcarnitine (C14), palmitoylcarnitine (C16) and stearoylcarnitine (C18) – showed significant differences between cases and controls (Table 2). Total amino acids showed a trend toward lower levels in the index cases compared with controls (*P*= 0.08), except alanine which showed a marginally significant difference (*P*= 0.05) (Table 3).

## Discussion

All 50 children enrolled in the study had classical T1D onset, diagnosed according to the ADA criteria,^9^ and 92% of them had detectable I-r-Abs or T1D HLA susceptibility haplotypes confirming the autoimmune nature of their T1D. After diagnosis, none of the children discontinued insulin replacement therapy either during the honeymoon period or during the 6 years of follow-up. As concerns the controls, for each single profile (circulating concentrations of carnitines and amino acids) of a ‘case' during the newborn screening test, four profiles present in the same analytic batch were reported as controls. Considering a potential bias, we can only state that, to date, no control has been reported to have developed T1D after 6 years of follow-up.

This is the first study to show that neonates with low levels of circulating carnitines and certain acyl-carnitines subsequently develop T1D (Figures 1a–c; Table 2). Although not significant, total amino acids showed a trend toward lower levels in cases vs controls (*P*=0.08) (Table 3). The points which reinforce the data obtained in our study are: (1) adequate number of index cases and appropriate choice of controls. The 50 neonates who subsequently developed T1D were matched with 200 controls, 4 for each of the index cases and born +/− 1 day; (2) a laboratory that since 2004 has enlarged the neonatal screening to 13 amino acids and to total carnitines, free carnitine and 33 acyl-carnitines; (3) the study was conducted in a completely blind fashion. By definition, the sole purpose of the neonatal screening until now has been to diagnose metabolic diseases in neonates, and not to predict T1D; (4) DBS are obtained within a few days of birth (48–72 h of life), so the blood extracted from them for measuring concentrations of amino acids and carnitines presents no problem of ‘decay' of the metabolites to be measured.^15^ It should be mentioned that the neonatal screening does not test the blood of the mother; (5) unlike other case-control studies, which cannot include an exposure/outcome (or risk/disease) time sequence, in this study the potential cause occurs before the disease, a necessary requisite for inferring a cause–effect relationship.

### The thymus and ‘central tolerance' at birth

There were two reasons why we wanted to know the levels of carnitine and its derivatives in the blood of the neonates who develop T1D. The first reason was that it had been reported that children and adolescents with overt T1D showed low levels of free carnitine in the blood, although circulating levels of acyl-carnitines were high.^7, 8^ Comparable data were obtained in experimental models^16, 17, 18^. However, we found that, at birth, our cohort of future T1D patients had significant low levels not only of free carnitine, but also of some acyl-carnitines.

The reason for these apparent differences is that our index cases, when tested at birth, were normal glycemic and not overt T1D. The second, and perhaps most relevant reason, is that the blood for neonatal screening is taken from neonates in the first days of life, a period crucial for the establishment of immunologic ‘central tolerance' in the thymus.^19^ In the first days of life, the thymic medulla is the site where ‘negative selection' of potentially autoreactive T cells takes place,^20^ and it is in the thymic cortex that ‘positive selection' of T cells occurs.^21^ When T cells are ‘positively selected' they exit the thymus and travel to the periphery, where they are responsible for defense against foreign agents such as viruses and bacteria.^22^ In those first few days of life, the generation of T regulatory (Treg) cells in the thymus is also in full swing.^23^

But, how does ‘negative selection' occur? Medullary thymic epithelial cells^24^ express almost all the tissue-specific autoantigens^25, 26^ which are then trimmed into 12–15 and 9 amino-acid peptides. The dodecapeptides occupy the grooves of the HLA class II molecules, whereas the nonapeptides occupy the grooves of the HLA class I molecules.^27^ The two HLA molecules combined with their respective peptides are inserted into the plasma membrane of the medullary thymic epithelial cells,^28^ which in turn expose these auto/self-peptides to potentially autoreactive CD4 and CD8 T cells. When the T-cell receptor of these potentially autoreactive CD4 and CD8 T cells encounters the auto/self-peptide in the grooves of the two HLA molecules expressed on the plasma membrane of the medullary thymic epithelial cells, the autoreactive T cells receive the ‘kiss of death' and more than 90% of them die by apoptosis in the first days of life.^29, 30^

### Carnitines, amino acids and ‘central tolerance' at birth

Carnitine (3-hydroxy-4-N-trymethylamonniobutanoate) is formed from methyl-lysine liberated from the body proteins and is an essential intracellular constituent for the transport of activated long chain fatty acids across membranes.^31^ Carnitine is biosynthesized in biologic cells and in tissues such as the human liver and brain in relatively high concentrations, either as free carnitine or as acyl-carnitines, including ALCAR. However, we do not yet know the degree to which systemic biomarkers match cellular pools. A significant amount of carnitine is obtained from the diet (mostly from milk products and red meat).^32^ Evidence exists that both the fetus and the placenta can synthesize carnitine,^33^ even though carnitine in the fetus is largely dependent on transplacental maternal carnitine transport rather than on endogenous synthesis.^34^

The next question is: how are low levels at birth of circulating carnitines linked to the etiopathogenesis of T1D? Let us first consider the role and function of ALCAR (C2 in Table 2). Pettegrew and collaborators^35^ pointed out that ALCAR is a potentially important biologic acetylating agent which could modify amino-acid and protein dynamics, function, turnover, structure and activity by acetylating –NH2 and –OH functional groups in amino acids such as lysine, serine, threonine and tyrosine as well as N-terminal amino acids.

Most tissue-specific autoantigens are hormones or hormone precursors, for example, insulin and thyroglobulin (Tg), or enzymes, for example, glutamic acid decarboxylase and thyroid peroxidase. Amino acids are important because they either feed the production of insulin and Tg or are the substrate for the expression of enzymes, for example, glutamic acid for glutamic acid decarboxylase and tyrosine for thyroid peroxidase. However, if at birth the levels of carnitines and amino acids are low, or poorly acetylated because of the low levels of ALCAR, their structure will not be modified and consequently their function will ultimately be impaired, tissue-specific autoantigens will not be expressed in the medullary thymic epithelial cells and consequently will not be trimmed, therefore the auto/self-peptides are not produced and ultimately the grooves of the HLA class I and II molecules will be occupied by other peptides.^36^ As a result of this, the T-cell receptor of potentially autoreactive T cells will not interact with the HLA-groove/auto/self-peptide complex, and thus 90% of the autoreactive T cells, that normally die in the first days of life, will survive and become ‘drifting mines' in the peripheral lymphoid organs, ready, if activated, to kill cells that are the potential target of an autoimmune attack, such as beta cells in the pancreatic islets. However, other mechanisms, including the weakening of Treg cells,^37^ or defective ‘peripheral tolerance'^38^ cannot be ruled out and may also be involved in the activation of autoreactive CD4 and CD8 T cells. Whether low levels of carnitines protect pancreatic beta cells from apoptosis is still debatable.

### Carnitines and human and experimental autoimmunity

It has been reported that Leghorn-type chicks fed after hatching with high doses of L-carnitine (1000 mg kg^−1^) have a significantly lower thymus weight relative to body weight.^39^ It remains to be shown whether the high dose of L-carnitine enhances the physiologic apoptotic process in the thymic medulla of these chicks. However, ALCAR has been shown to have an anti-apoptotic effect when incubated with several cell lines,^40^ but there are other reports which have indicated that, conversely, 2 acyl-carnitines, palmitoylcarnitine (C16)^41^ and 3-hydroxy-isovalerylcarnitine (C5OH),^42^ have a pro-apoptotic effect on certain cell lines. Interestingly, we have found that the levels of C5OH and C16 were significantly low at birth in our cohort of future T1D cases (Table 2). It could be argued that the low levels at birth of these acyl-carnitines certainly do not help the apoptotic process which eliminates autoreactive T cells in the thymic medulla, again in the critical first days of life when ‘negative selection' is more active. It has been observed in humans^43^ and in mice^44^ that carnitine has immunosuppressive/immunomodulating functions. Could it be that, in addition to C5OH or C16, the other acyl-carnitines, proprionylcarnitine (C3), miristoylcarnitine (C14) and stearoylcarnitine (C18), also found at low levels at birth (Table 2), are involved in particular immunologic functions in the thymus in the first days of life?

Apart from influencing ‘negative selection', low levels of carnitines may also have an effect on the functions of other thymic cell compartments, for example, those involved in the ‘positive selection' process,^16^ the generation of Treg cells^23^ or the functions of the thymic nurse cells, sets of cells which reside in the thymic cortex.^45, 46^ Obese strain chickens, which develop spontaneous autoimmune thyroiditis, are deficient in thymic nurse cells, the thymus containing only half the number of thymic nurse cells found in the thymus of normal white Leghorn chickens, beginning as early as at 17 days of embryogenesis.^47^ Would a high dose of carnitine, as given to Leghorn-type chicks at hatching, but given this time to obese brooding hens or to their chicks after hatching, restore the number and functions of thymic nurse cells?

What about non-obese diabetic (NOD) mice, the classic animal model of T1D? Although it has been debated whether ‘negative selection' in NOD mice is impaired,^48, 49^ it would be interesting to measure amino-acid and carnitine levels in newborn NOD mice to see if female NOD mice, which have a higher prevalence of T1D compared with male NOD mice, have lower levels of carnitines and amino acids at birth. If this is so, would giving a supplement of amino acids and carnitines to pregnant mothers or to neonatal NOD mice prevent them from developing T1D?

### Other relevant questions

What determines the levels of carnitines in neonates? The answer to this question might emerge from recent data showing that children 0–3 years of age and living in particular socioeconomic situations, including crowded homes or in families with poor nutritional conditions, have a threefold greater risk of developing T1D.^50^ Comparing their data with ours, it is interesting to note that 33 out of 50 (66%) of our index cases developed T1D between 0.8 and 3.8 years and 15 (30%) between 4.1 and 6.5 years (Table 1). Were the children in our T1D cohort living in similar conditions?

Could certain metabolic diseases enter into the T1D equation? In answer to this question, it is known that elevated concentrations of acyl-carnitines at birth are primary markers of fatty acid metabolic disorders (Table 4).^51^ CU/TDD (carnitine uptake/transport deficiency disorder) or SPDC (systemic primary carnitine deficiency) are exceptions, because low free carnitine levels have been found at birth in neonates who go on to develop a metabolic disorder characterized by cardiomyopathy and hypoglycemia.^52^ Despite the hypoglycemic episodes at birth, the level value of carnitine in our cohort of neonates who then went on to develop T1D was 17 μml l^−1^. In the light of our observations, it would be of interest to know if T1D will develop later in life in patients with CU/TDD–SPDC.

### Epigenetics, the ENCODE project and T1D

Epigenetic modifications, such as DNA methylation and post-translational histone modifications are indispensable for a variety of genomic processes, including maintenance of genomic integrity and regulation of gene expressions.^53^ By performing a genome-wide DNA methylation analysis,^54^ the presence of T1D-specific methylation variable positions (T1D-MVPs) was detected in 15 T1D monozygotic twins and in 7 islet cell antibody positive school children before and after they developed T1D. This report describes the first example of a disease associated with epigenetic modifications that antedate the onset of the clinical disease. It remains to be seen whether T1D-MVPs are potential etiologic factors for causing disease or rather are biomarkers. However, the relevance of these findings will certainly be reinforced if T1D-MVPs exist at birth and, if so, will support the possibility of a stochastic or environmental factor that takes effect in utero.^54^

Acetylation is one of the most frequent and better understood epigenetic changes in histones, neutralizing their charge and generating an open-chromatin configuration which allows the binding of transcription factors and chromatin-remodelling complexes that influence gene transcription.^4^ Even if we do not have evidence at the moment of histone acetylation which can confirm risk for T1D. We have already pointed out the relevant role of ALCAR in the acetylation of amino acids and proteins thereby giving them the right structure and proper activity. ALCAR, when detected at low levels at birth, may well be a potential etiologic factor, responsible for causing the disease, rather than being just a biologic marker for T1D-prediction. More relevantly, it might turn out to be the ‘spy' of the existence of environmental factors operating in utero, and thus responsible for low levels of carnitines at birth.

The initial results of the ENCODE project (Encyclopedia of DNA Elements) indicate that 80% of the genome contains elements linked to biochemical functions filled with enhancers (regulatory DNA elements), promoters (the sites at which DNA transcription into RNA is initiated) and numerous previously overlooked regions that encode RNA transcripts that are not translated into proteins, but that might have regulatory roles.^55^ These results show that many DNA variants previously correlated with certain diseases lie within or very near non-coding functional DNA elements, providing new leads for linking epigenetic variations and disease. T1D-MVPs and our own data on the at-birth low levels of carnitines, both of which involve methylation and acetylation variations, may now provide the right insights for dissecting the etiopathogenesis of T1D.

Of note, poor acetylation of amino acids and proteins at birth will be a risk factor for developing T1D and other autoimmune diseases, such as autoimmune thyroid disease^56^ and CD^57^ (CD was found primarily associated with T1D in our cohort), but it must be pointed out that the ‘risk' applied only in HLA genetically susceptible individuals (for example, HLA DR3/DR4) and not in individuals with other HLA haplotypes.

## Conclusion

In conclusion, neonates that might develop T1D later in life have reduced levels, soon after birth, of circulating free carnitine and acyl-carnitines and a trend toward low levels of amino acids. Laboratory determination of these compounds is easy to perform and is routinely applied in neonatal mass screenings. The evaluation of amino-acid concentrations and carnitines in DBS could therefore represent an additional tool for HLA genotyping (HLA DR3/DR4) to predict T1D and may also give rise to new strategies for the primary prevention of T1D as early as from birth. At this point, a clinical trial, where HLA at ‘risk' newborns will be bottle-fed with a supplementation of these nutrients or metabolites for a few weeks after birth, could be organized to prove or disprove whether the primary prevention of T1D is feasible.

## Figures and Tables

**Table 1:** Clinical, familial, immunological, genetic and metabolic characteristic of the 50 children who developed T1D

*Initials*	*Sex*	*Date of birth*	*Date of T1D onset*	*Age of onset of T1D*	*Family members with T1D and other AI deseases*	*Beta cell autoimmunity*	*Associated diseases*	*Date of associated disease diagnosis*	*Islet cell antibodies (ICA;JDF/U)*	*Insulin autoantibodies (IAA;UL ml^−1^)*	*Glutaminc acid decarboxylase antibodies (GADA;UL ml^−1^)*	*Insulinoma associated-antigen 2 antibodies (IA2A;UL ml^−1^)*	*HLA-DR*	*HLA-DQ*	*pH*	*Glycemia (mg dl^−1^)*	*Glycosylated Hemoglobin %*	*C-peptide*
LA	F	31/08/2005	18/02/2007	1.6	father T1D	Yes	CD	03/06/2009	Neg	5	0.7	0.5	NT	NT	7.17	555	9.3	0.58
CE	M	05/08/2008	01/07/2009	0.9	No	Yes	No		Neg	4	0.7	<0.5	4.13	3.6	6.9	1243	10.4	NK
CGF	M	14/03/2006	11/02/2008	1.9	Father Graves' disease	Yes	No		Neg	NT	24.5	0.7	1	5	6.99	483	11.1	0.18
GI	F	01/12/2008	09/11/2009	0.9	No	Yes	CD	24/10/2011	5	NT	20.4	<0.5	NT	NT	7.27	643	7.3	NK
TT	M	05/02/2006	24/12/2007	1.9	No	Yes	No		80	4.4	<0.3	<0.5	4.17	2.8	7.32	490	7.7	NK
GP	M	05/06/2007	26/07/2011	4.1	No	Yes	CD	24/04/2012	NT	4	<0.3	12.3	NT	NT	7.39	427	8.3	NK
GM	F	13/04/2010	22/02/2012	1.9	No	Yes	CD	24/05/2012	>350	NT	>350	NT	4.15	3.6	7.07	456	8.4	0.4
AD	F	28/04/2011	25/01/2012	0.8	No	Yes	No		>5	3	148	Neg	3.4	2.3	6.82	750	8.6	NK
DA	M	20/08/2008	26/10/2010	2.2	Father T1D	Yes	No		Neg	NT	<0.3	18.2	4.13	3.6	7.28	700	8.7	0.27
PNA	M	05/11/2008	26/02/2012	3.3	No	Yes	No		NT	NT	<0.1	4.1	NT	NT	7.31	592	8.7	<0.7
IS	M	15/01/2006	16/06/2010	4.5	Mother CD	Yes	CD	25/10/2010	40	NT	NT	NT	3.4	2.3	7.39	521	8.8	0.33
LR	M	18/07/2009	28/03/2011	1.8	Sister T1D	Yes	CD	01/09/2012	NT	Neg	<0.3	7.4	4	3	7.01	677	9	<0.1
SD	M	20/12/2005	12/09/2008	2.9	No	No	No		<5	Neg	<0.3	<0.5	3.4	2.3	6.9	589	9	0.34
MM	F	07/02/2006	31/10/2009	3.8	No	Yes	No		NT	8	2.5	43.8	1.4	1.3	7.4	345	9.1	0.13
CA	M	05/11/2006	09/02/2012	5.4	NK	NT	NK		NT	NT	NT	NT	NT	NT	7.37	501	9.1	<0.8
RA	F	16/05/2008	02/10/2010	2.5	No	Yes	CD	20/08/2012	10	Neg	9.7	1.65	NT	NT	7.01	468	9.2	0.7
FG	M	16/06/2007	30/01/2009	1.7	No	Yes	No		10	NT	0.3	9.2	1.4	3.5	7.25	312	9.5	0.1
AA	F	27/03/2006	19/12/2010	4.8	Father T1D	Yes	CD	10/07/2011	5	NT	15	21.8	4.11	3.3	7.31	549	9.6	0.5
PA	F	25/07/2007	04/05/2012	4.9	No	Yes	No		>5	NT	54.4	0.5	4.13	2.6	7.34	440	9.6	0.74
PC	F	02/08/2010	12/07/2011	0.9	No	Yes	No		NT	Pos	32.8	1	NT	NT	6.84	530	9.8	NK
NN	M	19/04/2010	29/10/2011	1.5	No	NT	NK		NT	NT	NT	NT	NT	NT	6.99	403	9.9	0.2
BT	M	26/12/2004	12/03/2008	3.3	No	Yes	No		30	NT	1.5	0.6	1.16	5	7.29	585	10	0.23
ZM	F	29/04/2007	03/03/2009	1.9	No	Yes	No		25	NT	NT	NT	3	2	7.23	870	10.2	0.32
RM	F	12/07/2005	08/02/2010	4.7	No	Yes	No		NT	4.2	21.3	NT	NT	NT	7.37	595	10.2	0.35
FF	F	22/05/2007	14/12/2011	4.7	No	Yes	No		<5	NT	81	0.9	3.4	2.3	7.4	487	10.4	NK
TA	F	21/06/2006	18/03/2011	4.9	No	NT	CD	10/10/2009	NT	NT	NT	NT	NT	NT	7.37	660	10.5	NK
FS	F	16/12/2005	03/02/2008	2.2	Grandmother T1D	Yes	No		NT	NT	2.1	10.8	NT	NT	7.25	616	10.9	NK
ME	M	15/03/2005	26/11/2006	1.8	No	Yes	No		NT	3.5	11.5	3.6	NT	NT	7.08	508	11.1	<0.5
MS	F	20/12/2005	05/04/2012	6.5	No	Yes	No		Neg	NT	2.1	7.03	NT	NT	7.22	555	11.4	0.22
ST	M	04/09/2006	31/01/2012	5.4	No	Yes	No		NT	NT	3.15	NT	NT	NT	7.34	626	11.4	0.16
LBAM	F	10/06/2008	28/09/2009	1.3	No	Yes	No		40	NT	NT	NT	NT	NT	6.97	582	12	NK
SDF	F	08/10/2006	18/04/2011	4.6	No	Yes	No		NT	3	15	5.1	3.4	2.3	7.3	439	12.1	<0.41
SS	F	10/09/2005	23/09/2007	2	No	NT	CD	01/05/2008	Neg	NT	NT	NT	4.7	2.8	7.21	635	12.9	NK
LS	F	04/01/2006	30/11/2008	2.9	No	Yes	No		Pos	NT	<0.1	5.9	NT	NT	7.18	743	12.9	0.3
MHME	M	18/12/2005	09/12/2011	6	No	Yes	No		NT	1.5	<0.5	NT	4.9	2.3	7.4	277	14	NK
BF	M	22/09/2008	27/08/2009	0.9	No	Yes	No		40	4	43.5	<0.5	3.4	2.3	7.05	439	12	NK
EOA	F	08/10/2009	23/07/2011	1.9	No	Yes	No		NT	3	5.2	16.3	3.13	2.3	7.35	937	11	1.14
PG	F	03/06/2005	15/12/2009	4.5	No	Yes	No		80	NT	27.8	9.3	3.16	2.5	7.34	535	11.8	NK
FC	F	02/07/2008	12/12/2009	1.5	Mother and grandmother T1D	Yes	No		5	NT	4.7	<0.5	3.4	2.3	7.39	312	9.1	NK
BF	M	10/02/2005	.06/11/2007	2.9	No	Yes	No		13.75	NT	98	NT	3.4	2.3	7.32	648	12.7	0.18
PG	M	18/09/2006	16/08/2009	3.1	No	Yes	No		5	1.7	<0,3	2.3	3.4	2.3	6.98	567	13.2	0.18
BR	M	24/07/2006	18/09/2009	3.2	Father T1D	Yes	No		<5	NT	<0,3	2.8	3.4	2.3	7.38	200	8.9	NK
BA	F	21/12/2004	03/05/2010	5.5	No	Yes	CD	24/11/2010	NT	1	32.9	30.3	4.7	2.3	7.29	412	10.5	0.19
AO	M	17/01/2005	.25/09/2009	4.8	No	Yes	No		NT	NT	7.2	38	4.8	3	7.23	514	13.7	NK
CDCD	F	20/12/2008	13/12/2009	1	No	Yes	No		40	NT	NT	NT	4.9	3.3	6.86	849	14	NK
UM	M	17/08/2008	08/04/2010	1.8	No	NT	No		NT	NT	NT	NT	NT	NT	7.21	655	12.8	NK
GE	M	16/07/2006	12/10/2011	5.3	No	Yes	No		<5	6.3	92.5	5.2	NT	NT	7.29	617	11.4	NK
VM	F	08/11/2005	17/12/2010	5.1	No	Yes	No		Neg	NT	25.7	5.5	NT	NT	7.45	663	10.9	NK
GM	F	15/02/2005	01/02/2007	2	No	Yes	No		NT	NT	65.5	<0.7	NT	NT	7.08	471	13.2	NK
PM	M	07/05/2007	13/05/2009	2	No	Yes	No		20	NT	NT	NT	NT	NT	7.01	612	12.7	NK

Abbreviations: CD, celiac disease; NK, not known; NT, not tested; T1D, type 1 diabetes.

**Table 2:** Carnitine fractions in cases and controls (μmol l^−1^)

	*Cases*	*Controls*	P*-value*
Total carnitine^a^	55.01 (12.16)	62.78 (15.68)	<0.001
Free carnitine^b^	22.38 (6.61)	26.51 (8.67)	0.002
acyl-carnitine^a^	32.63 (7.42)	36.27 (8.80)	0.007
Acyl/free carnitine ratio^a^	1.53 (0.38)	1.43 (0.33)	0.07
C2^a^ acetyl-L-carnitine (ALCAR)	24.28 (6.18)	26.86 (7.17)	0.02
C3^b^ proprionylcarnitine	1.27 (0.47)	1.54 (0.79)	0.002
C5OH^a^ hydroxyisovalerylcarnitine	0.10 (0.03)	0.12 (0.04)	0.004
C14^b^ miristoylcarnitine	0.19 (0.09)	0.21 (0.10)	0.037
C16^b^ palmitoylcarnitine	2.32 (1.06)	2.48 (1.23)	0.038
C18^b^ stearoylcarnitine	0.71 (0.34)	0.74 (0.33)	0.043

Mann–Whitney rank test for *P*-value.

a Normal distribution: values are means (standard deviations); Student's *t*-test for *P*-value.

b Non-normal distribution: values are medians (25th–75th interquartile ranges); rank test for *P*-value.

**Table 3:** Amino acids in cases and in controls (μmol l^−1^)

	*Diabetes*	*Controls*	P*-value*
Alanine^a^	134.82 (37.95)	147.72 (42.45)	0.05
Phenylalanine^a^	41.83 (13.98)	43.84 (14.52)	0.37
Valina^b^	104.81 (45.58)	108.31 (46.44)	0.29
Leucine-Isoleucine^b^	95.89 (38.54)	97.26 (33.44)	0.28
Metionina^b^	16.12 (7.30)	16.35 (5.95)	0.48
Tyrosine^b^	53.16 (32.78)	54.14 (28.31)	0.88
Aspartate^b^	29.67 (17.52)	30.41 (17.83)	0.57
Ornitine^b^	26.63 (9.64)	26.29 (10.44)	0.78
Glutamate^b^	250.30 (104.84)	276.13 (124.34)	0.20
Glycine^a^	338.71 (97.29)	354.17 (93.44)	0.30
Arginine^b^	5.07 (3.48)	5.45 (4.31)	0.65
Cytosine^b^	8.09 (2.72)	8.38 (3.31)	0.40
Argininosuccinate^b^	0.20 (0.45)	0.21 (0.35)	0.99
Total amino acids	1151.76 (218.21)	1212.68 (218.73)	0.08

Mann–Whitney rank test for *P*-value.

a Normal distribution: values are means (standard deviations); Student's *t*-test for *P*-value.

b Non-normal distribution: values are medians (25th–75th interquartile ranges).

**Table 4:** Fatty acid metabolism disorders^a^ (°=Primary markers)

Medium chain acyl co-A dehydrogenase deficency (MCAD)
°Elevated C8 (octanooyl carnitine)
°Elevated C8/C10

Medium chain ketoacyl co-A thiolase deficency
°Elevated C8 (octanooyl carnitine)
°Elevated C6DC (adpyl carnitine)

Short chain acyl co-A dehydrogenase deficency (SCAD)
°Elevated C4 (butyryl carnitine)

Medium and short chain acyl co-A dehydrogenase deficency (M/SCHAD)
°Elevated C4OH (3-OH butyryl carnitine)

Dienoyl co-A reductase deficency
°Elevated C10:2 (decadienoyl carnitine)

Long chain 3-OH acyl co-A dehydrogenate deficency (LCHAD)
°Elevated C16-OH (3-OH palmitoyl carnitine)

Very long chain acyl co-A dehydrogenase deficency (VLCAD)
°Elevated C14:1 (tetradecenoyl carnitine)
°Elevated C14:1/C12:1
°Elevated C14:1/C16

Acyl co-A dehydrogenase deficency /glutaric acidurin type II (MAD/GA II)
°Elevated C4 (butyryl carnitine)
°Elevated C5 (isovaleryl carnitine)

**Carnitine uptake/transport deficency**
**°Low free carnitine**

Carnitine palmitoyl transferase I deficency (CPT I)
°Elevated free carnitine/C16 + C18

Carnitine palmitoyl transferase II deficency (CPT II)
°Elevated C16 (palmitoyl carnitine)
°Elevated C18:1 (oleyl carnitine)

Carnitine/acylcarnitine translocase deficency (CACT)
°Elevated C16 (palmitoyl carnitine)
°Elevated C18:1 (oleyl carnitine)

a Fatty acids metabolic disorders show elevated levels of certain acyl-carnitines at birth, but in neonates with Carnitine Uptake/Transport deficency (CU/TD), the levels of free carnitine are low (see CU/TD highlighted in the table). At the light of our findings of low levels of free carnitine in neonates who then developed T1D, we wonder whether children affected by CU/TD develop T1D more often than expected.

## References

[bib1] Todd J Etiology of type 1 diabetes Immunity 2010 32 457 467 20412756 10.1016/j.immuni.2010.04.001

[bib2] La Torre D Lernmark Ä Immunology of β-cell destruction Adv Exp Med Biol 2010 654 537 583 20217514 10.1007/978-90-481-3271-3_24

[bib3] Vehik K Dabelea D The changing epidemiology of type 1 diabetes: why is it going through the roof Diabetes Metab Res Rev 2010 27 3 13 21218503 10.1002/dmrr.1141

[bib4] Jayaraman S Epigenetics of autoimmune diabetes Epigenomics 2011 3 639 648 22126251 10.2217/epi.11.78

[bib5] Knip M Virtanen SM Becker D Dupré J Krischer JP Åkerblom HK TRIGR Study Group Early feeding and risk of type 1 diabetes: experiences from the trial to reduce insulin-dependent diabetes mellitus in the Genetically at Risk (TRIGR) Am J Clin Nutr 2011 94 (6 Suppl 1814S 1820S 21653795 10.3945/ajcn.110.000711 PMC3364078

[bib6] Snell-Bergeon JK Smith J Dong F Barón AE Barriga K Norris JM Early childhood infections and the risk of islet autoimmunity: the diabetes autoimmunity study in the young (DAISY) Diabetes Care 2012 35 2553 2558 23043167 10.2337/dc12-0423 PMC3507568

[bib7] Coker M Coker C Darcan S Can S Orbak Z Göksen D Carnitine metabolism in diabetes mellitus J Pediatr Endocrinol Metab 2002 15 841 849 12099395 10.1515/jpem.2002.15.6.841

[bib8] Mamoulakis D Galanakis E Dionyssopoulou E Evangeliou A Sbyrakis S Carnitine deficiency in children and adolescents with type 1 diabetes J Diabet Complications 2004 18 271 274 10.1016/S1056-8727(03)00091-6 15337500

[bib9] American Diabetes Association Diagnosis and classification of diabetes mellitus Diabetes Care 2012 35 (Suppl 1 S64 S71 22187472 10.2337/dc12-s064 PMC3632174

[bib10] Olerup O Zetterquist H HLA-DR typing by PCR amplification with sequence-specific primers (PCR-SSP) in 2 h: an alternative to serological DR typing in clinical practice including donor-recipient matching in cadaveric transplantation Tissue Antigens 1992 39 225 235 1357775 10.1111/j.1399-0039.1992.tb01940.x

[bib11] la Marca G Malvagia S Pasquini E Innocenti M Donati MA Zammarchi E Rapid diagnosis of medium chain acyl Co-A dehydrogenase (MCAD) deficiency in a newborn by liquid chromatography/tandem mass spectrometry Rapid Commun Mass Spectrom 2003 17 2688 2692 14648909 10.1002/rcm.1248

[bib12] la Marca G Malvagia S Pasquini E Innocenti M Donati MA Zammarchi E Rapid 2nd-tier test for measurement of 3-OH-propionic and methylmalonic acids on dried blood spots: reducing the false-positive rate for propionylcarnitine during expanded newborn screening by liquid chromatography-tandem mass spectrometry Clin Chem 53 1364 1369 2007 17510301 10.1373/clinchem.2007.087775

[bib13] la Marca G Malvagia S Pasquini E Innocenti M Fernandez MR Donati MA The inclusion of Succinylacetone as marker for Tyrosinemia type I in expanded newborn screening programs Rapid Commun Mass Spectrom 2008 22 812 818 18278819 10.1002/rcm.3428

[bib14] Azzari C la Marca G Resti M Neonatal screening for severe combined immunodeficiency caused by an adenosine deaminase defect: a reliable and inexpensive method using tandem mass spectrometry J Allergy Clin Immunol 2011 127 1394 1399 21624616 10.1016/j.jaci.2011.03.040

[bib15] la Marca G Malvagia S Casetta B Pasquini E Donati MA Zammarchi E Progress in expanded newborn screening for metabolic conditions by LC-MS/MS in Tuscany: update on methods to reduce false tests J Inherit Metab Dis 2008 31 (Suppl 2 S395 S404 18956250 10.1007/s10545-008-0965-z

[bib16] Bin Aleem S Hussain MM Farooq Y Serum levo-carnitine levels and skeletal muscle functions in type 2 diabetes mellitus in rodents J Coll Physicians Surg Pak 2013 23 132 136 23374518

[bib17] Hao Y Basile AS Chen G Zhang L Glutamate-induced over-expression of GAD is down-regulated by acetyl-L-carnitine in rat islet cells Endocr Res 2004 30 107 116 15098924 10.1081/erc-120029890

[bib18] Cresto JC Fabiano de Bruno LE Cao GF Pastorale CF Confalonieri N del Carmen Camberos M The association of acetyl-L-carnitine and nicotinamide remits the experimental diabetes in mice by multiple low-doses streptozotocin Pancreas 2006 33 403 411 17079947 10.1097/01.mpa.0000236740.07854.b1

[bib19] Klein L Hinterberger M Wirnsberger G Kyewski B Antigen presentation in the thymus for positive selection and central tolerance induction Nat Rev Immunol 2009 9 833 844 19935803 10.1038/nri2669

[bib20] Kappler JW Roehm N Marrack P T cell tolerance by clonal elimination in the thymus Cell 1987 49 273 280 3494522 10.1016/0092-8674(87)90568-x

[bib21] Kisielow P Teh HS Blüthmann H von Boehmer H Positive selection of antigen-specific T cells in thymus by restricting MHC molecules Nature 1988 335 730 733 3262831 10.1038/335730a0

[bib22] Yewdell JW Dolan BP Immunology: Cross-dressers turn on T cells Nature 2011 471 581 582 21455165 10.1038/471581a PMC3400133

[bib23] Klein L Jovanovic K Regulatory T cell lineage commitment in the thymus Semin Immunol 2011 23 401 409 21733719 10.1016/j.smim.2011.06.003

[bib24] Derbinski J Schulte A Kyewski B Klein L Promiscuous gene expression in medullary thymic epithelial cells mirrors the peripheral self Nat Immunol 2001 2 1032 1039 11600886 10.1038/ni723

[bib25] Geenen V Legros JJ Franchimont P BaurdiHaye M Defresne MP Boniver J The neuroendocrine thymus: coexistence of oxytocin and neurophysin in the human thymus Sience 1986 253 508 511 10.1126/science.3961493 3961493

[bib26] Derbinski J Gäbler J Brors B Tierling S Jonnakuty S Hergenhahn M Promiscuous gene expression in thymic epithelial cells is regulated at multiple levels J Exp Med 2005 202 33 45 15983066 10.1084/jem.20050471 PMC2212909

[bib27] Yaneva R Schneeweiss C Zacharias M Springer S Peptide binding to MHC class I and II proteins: new avenues from new methods Mol Immunol 2010 47 649 657 19910050 10.1016/j.molimm.2009.10.008

[bib28] Vyas JM Van der Veen AG Ploegh HL The known unknowns of antigen processing and presentation Nat Rev Immunol 2008 8 607 618 18641646 10.1038/nri2368 PMC2735460

[bib29] Surh CD Sprent J T-cell apoptosis detected *in situ* during positive and negative selection in the thymus Nature 1994 372 100 103 7969401 10.1038/372100a0

[bib30] Medzhitov R Janeway CA Jr. How does the immune system distinguish self from nonself Semin Immunol 2000 12 185 188 10910738 10.1006/smim.2000.0230

[bib31] Bremer J The role of carnitine in intracellular metabolism J Clin Chem Clin Biochem 1990 28 297 301 2199593

[bib32] Carter AL Abney TO Lapp DF Biosynthesis and metabolism of carnitine J Child Neurol 1995 2 S3 S7 8576566

[bib33] Oey NA van Vlies N Wijburg FA Wanders RJA Attie-Bitach T Vaz FM L-carnitine is synthesized in the human fetal-placental unit: potential roles in placental and fetal metabolism Placenta 2006 27 841 846 16300828 10.1016/j.placenta.2005.10.002

[bib34] Vijay S Patterson A Olpin S Henderson MJ Clark S Day C Carnitine transporter defect: diagnosis in asymptomatic adult women following analysis of acyl-carnitines in their newborn infants J Inherit Metab Dis 2006 29 627 630 16865412 10.1007/s10545-006-0376-y

[bib35] Pettegrew JW Levine J McClure RJ Acetyl-L-carnitine physical-chemical, metabolic, and therapeutic properties: relevance for its mode of action in Alzheimer's disease and geriatric depression Mol Psychiatry 2000 5 616 632 11126392 10.1038/sj.mp.4000805

[bib36] Neefjes J Jongsma ML Paul P Bakke O Towards a systems understanding of MHC class I and MHC class II antigen presentation Nat Rev Immunol 2011 11 823 836 22076556 10.1038/nri3084

[bib37] Olivieri A De Angelis S Dionisi S D'Annunzio G Locatelli M Marinaro M Serum transforming growth factor β1 during diabetes development in non-obese diabetic mice and humans Clin Exp Immunol 2010 162 407 414 20819089 10.1111/j.1365-2249.2010.04253.x PMC3026544

[bib38] Zehn D Bevan MJ T cells with low avidity for a tissue-restricted antigen routinely evade central and peripheral tolerance and cause autoimmunity Immunity 2006 25 261 270 16879996 10.1016/j.immuni.2006.06.009 PMC2774714

[bib39] Deng K Wong CW Nolan JV Long-term effects of early-life dietary L-carnitine on lymphoid organs and immune responses in Leghorn-type chickens J Anim Physiol Anim Nutr (Berl) 2006 90 81 86 16422773 10.1111/j.1439-0396.2005.00569.x

[bib40] Chapela SP Kriguer N Fernández EH Stella CA Involvement of L-carnitine in cellular metabolism: beyond Acyl-CoA transport Mini Rev Med Chem 2009 9 1518 1526 20205633 10.2174/138955709790361502

[bib41] Mutomba MC Yuan H Konyavco M Adachi S Yokoyama CB Esser V Regulation of the activity of caspases by L-carnitine and palmitoylcarnitine FEBS Lett 2000 487 19 25 10922462 10.1016/s0014-5793(00)01817-2

[bib42] Ferrara F Bertelli A Falchi M Evaluation of carnitine, acetylcarnitine and isovalerylcarnitine on immune function and apoptosis Drugs Exp Clin Res 2005 31 109 114 16033249

[bib43] Fortin G Yurchenko K Collette C Rubio M Villani AC Bitton A L-carnitine, a diet component and organic cation transporter OCTN ligand, displays immunosuppressive properties and abrogates intestinal inflammation Clin Exp Immunol 2009 156 161 171 19175620 10.1111/j.1365-2249.2009.03879.x PMC2673754

[bib44] Athanassakis I Mouratidou M Sakka P Evangeliou A Spilioto M Vassiliadis S L-carnitine modifies the humoral immune response in mice after *in vitro* or *in vivo* treatment Int Immunopharmacol 2001 1 1813 1822 11562072 10.1016/s1567-5769(01)00105-9

[bib45] Wekerle H Ketelsen UP Ernst M Thymic nurse cells. Lymphoepithelial cell complexes in murine thymuses: morphological and serological characterization J Exp Med 1980 151 925 944 6966312 10.1084/jem.151.4.925 PMC2185829

[bib46] Hendrix TM Chilukuri RV Martinez M Olushoga Z Blake A Brohi M Thymic nurse cells exhibit epithelial progenitor phenotype and create unique extra-cytoplasmic membrane space for thymocyte selection Cell immunol 2010 261 81 92 20035931 10.1016/j.cellimm.2009.11.004 PMC2830717

[bib47] Boyd RL Oberhuber G Hála K Wick G Obese strain (OS) chickens with spontaneous autoimmune thyroiditis have a deficiency in thymic nurse cells J Immunol 1984 132 718 724 6361133

[bib48] Liston A Lesage S Gray DH O'Reilly LA Strasser A Fahrer AM Generalized resistance to thymic deletion in the NOD mouse; a polygenic trait characterized by defective induction of Bim Immunity 2004 21 817 830 15589170 10.1016/j.immuni.2004.10.014

[bib49] Mingueneau M Jiang W Feuerer M Mathis D Benoist C Thymic negative selection is functional in NOD mice J Exp Med 2012 209 623 637 22329992 10.1084/jem.20112593 PMC3302233

[bib50] Bruno G Spadea T Picariello R Gruden G Barutta F Cerutti F Early life socioeconomic indicators and risk of type 1 diabetes in children and young adults J Pediatr 2013 162 600 605 23084710 10.1016/j.jpeds.2012.09.010

[bib51] Rinaldo P Matern D Bennett MJ Fatty acid oxidation disorders Annu Rev Physiol 2002 64 477 502 11826276 10.1146/annurev.physiol.64.082201.154705

[bib52] Magoulas PL El-Hattab AW Systemic primary carnitine deficiency: an overview of clinical manifestations, diagnosis, and management Orphanet J Rare Dis 2012 18 68doi:10.1186/1750-1172-7-68 22989098 PMC3495906

[bib53] Jaenisch R Bird A Epigenetic regulation of gene expression: how the genome integrates intrinsic and environmental signals Nat Genet 2003 33 (Suppl 245 254 12610534 10.1038/ng1089

[bib54] Rakyan VK Beyan H Down TA Hawa MI Maslau S Aden D Identification of type 1 diabetes-associated DNA methylation variable positions that precede disease diagnosis PLoS Genet 2011 7 e1002300 21980303 10.1371/journal.pgen.1002300 PMC3183089

[bib55] Ecker JR Bickmore WA Barroso I Pritchard JK Gilard Y Segal E Genomics: ENCODE explained Nature 2012 489 52 55 22955614 10.1038/489052a

[bib56] Lenzi L Mirri S Generoso M Guasti M Barni F Pepe R Thyroid autoimmunity and type 1 diabetes in children and adolescents: screening data from Juvenile Diabetes in Tuscany Regional Centre Acta Biomed 2009 80 203 206 20578412

[bib57] Camarca ME Mozzillo E Nugnes R Zito E Falco M Fattorusso V Celiac disease in type 1 diabetes mellitus Ital J Pediatr 2012 38 10doi:10.1186/1824-7288-38-10 22449104 PMC3348012

